# Quantifying the portrayal of alcohol-related A&E attendances and prevention in the British medical documentary series ‘24 hours in A&E’

**DOI:** 10.1093/pubmed/fdae314

**Published:** 2025-01-12

**Authors:** Karielle Webster, Holly Knight, Joanne R Morling

**Affiliations:** Lifespan and Population Health, School of Medicine, University of Nottingham, Nottingham NG5 1PB, UK; Lifespan and Population Health, School of Medicine, University of Nottingham, Nottingham NG5 1PB, UK; Lifespan and Population Health, School of Medicine, University of Nottingham, Nottingham NG5 1PB, UK; NIHR Nottingham Biomedical Research Centre (BRC), Nottingham University Hospitals NHS Trust and the University of Nottingham, Nottingham NG7 2UH, UK

**Keywords:** alcohol consumption, population-based and preventative services, health promotion

## Abstract

**Background:**

Alcohol misuse is linked to numerous health and socioeconomic harms. Edutainment and docutainment television programmes can act as health promotion tools, influencing health perceptions and behaviours. Inaccurate portrayals can engender misinformation. Limited research has assessed alcohol-related illnesses and prevention in edutainment/docutainment, with none examining British medical documentaries.

**Methods:**

A quantitative content analysis assessed the portrayal of alcohol-related attendances (ARAs), behaviours, and prevention in the series *24 hours in A&E*. Main series episodes broadcast 2011–2022, depicting ARAs, were coded. Descriptive statistics and a Fisher’s exact were then undertaken.

**Results:**

ARAs featured 38 patients in 23 episodes (8.3% episodes total). Significantly more ARA episodes were broadcast from 2011 to 2016 than 2017–2022 (*P* = 0.002). ARAs were mainly portrayed in males (63.2%), young adults (73.7%) and White ethnic groups (78.9%). Binge drinking and pubs/bar/nightclubs/‘nights out’ were the main behaviours and settings depicted. ARAs encompassed predominantly accidents/injuries (72.7%). Prevention featured infrequently (15.8% patients) and involved secondary (50.0%) or tertiary prevention (50.0%) for alcohol-use disorders (AUDs).

**Conclusion:**

ARAs were under-portrayed. While prevention portrayals and demographics were largely consistent with reality, ethnic minority groups, AUDs and chronic alcohol-related illnesses were underrepresented. Binge drinking and night-time economy settings were over-portrayed. Inaccurate depictions could lead to misperceptions of alcohol-related health harms.

## Introduction

Alcohol accounts for an estimated 3 million deaths each year and 5.0% of the total burden of disease worldwide.^1^ While alcohol use is deeply entrenched in British culture,^2^ often being synonymous with positive aspects of life,^3^ research suggests alcohol contributes to >70% of weekend accident and emergency (A&E) attendances.^4^

The UK screening and brief intervention (SBI) model^5^^,^^6^ is recommended for use by A&E clinicians and represents the mainstay of alcohol prevention activities undertaken in these settings.^7^^,^^8^ A&E settings are well placed to deliver SBI^9^ as they see a high volume of patients with alcohol-related attendances (ARAs) and unhealthy drinking behaviours.^6^

The media (e.g. television (TV), print, radio) and social media are important sources of the public’s health information,^10–12^ which can convey both health-promoting and health-damaging messaging.^13^^,^^14^ It shapes people’s health perceptions, behaviours and healthcare service use by setting the agenda on which health issues are considered important and how these are framed.^13^^,^^14^

Medical documentaries can span the boundaries of reality TV, documentary, entertainment and educational programming.^15–18^ Medical documentaries generally depict ‘real’ people’s illness journeys and interactions with healthcare professionals, interspersed with health statistics and clinician accounts.^19^

Medical docutainment and edutainment programmes typically present selective reality and can reach large audiences.^20^ They are deemed trustworthy and believable sources of health information by viewers.^21^^,^^22^ In addition to influencing health knowledge, attitudes and behaviours,^20^ medical docutainment and edutainment can shape perceptions of health conditions,^23^ clinicians^22^ and treatments.^24^

There are concerns that medical docutainment and edutainment programmes overrepresent more extreme, rare and dramatic illnesses, while under-portraying common real-life conditions, such as non-communicable diseases.^25–27^ This study aimed to explore the portrayal of ARAs and prevention in the British medical docutainment TV series, *24 Hours in A&E*, with a sociodemographic patient characteristic focus, using a quantitative content analysis.

## Methods

*24 Hours in A&E* is an award-winning British medical documentary/docutainment series, set initially in two London hospitals. Each episode follows patients treated in the hospital’s A&E department over a single 24-hour period. The programme was selected for this study due to its long run,^28^ popularity (ranked 60/100 most popular contemporary TV series in the UK)^29^ and setting in a busy, urban A&E environment.

### Sample and data sources

In total, 300 episodes across 26 series of *24 Hours in A&E* were broadcast between 2011 and 2022 (Supplementary material Table S1). Series 1 first aired in May 2011 and series 26 (most recent at the time of this study) in January 2022.

Episode synopses were reviewed on the Internet Movie Database (IMDb)^30^ and Wikipedia to identify relevant episodes portraying ARAs. Episodes featuring ARAs were defined as those where the synopsis referenced attendances associated with alcohol use and/or settings where alcohol was likely to be consumed. Twenty-two ‘special’ episodes, which focused on specific diseases or issues, were excluded as none featured alcohol as a theme.

### Unit of analysis

Full episodes were used for coding. The unit of analysis was an episode-functional individual adult patient ‘character’. This was defined as a patient (aged 18 years or more) who visually featured in an episode for a significant period as a main or supporting ‘character’ in the episode’s ‘plotline’, was seen on multiple occasions and/or reoccurred during most of an episode. Patients who were only seen briefly or referenced verbally were not coded.

Patient ‘characters’ were categorized as main ‘characters’, if their story was a major focus of an episode, or supplementary, if they featured in a supporting context.

### Measures

Coding for the variables assessed was determined based on the on-screen portrayals of patient-clinician consultations and/or wider episode narratives.

Demographic data was coded as stated in the episode, or the author’s visual assessment and judgement:

#### Age group

Young adult (18–35 years), middle aged (36–64 years) or older adult (aged 65 years and over).

#### Sex

Externally presented as male or female.

*Ethnicity* was externally ascribed and classified broadly based on the main aggregated ethnicity categories used in the 2021 England Census.^31^

ARAs were categorized according to whether the patient had consumed alcohol in the context of their A&E attendance, and/or their presentation involved a setting where alcohol was likely to be consumed (explicitly stated or implied), either by the patient themselves or others.

#### Alcohol-related setting

Main type of setting involved in the patient’s A&E attendance as pubs/bars/clubs/‘nights out’, private parties, restaurants or other type of setting related to the night-time economy or combined.

#### Alcohol use

Where alcohol use was explicitly mentioned in the context of a patient’s attendance, the primary drinking behaviour type was coded. Categorization was based on National Institute for Health and Care Excellence (NICE) classifications for alcohol dependence, binge drinking, lower, increasing or high-risk drinking behaviours.^7^ High-risk drinking also referred to as harmful drinking, encompasses consuming 35 units/week for women and >50 units/week for men.^7^ Drinking behaviours were categorized based on specific unit amounts, quantities of alcohol consumed or risk categories mentioned in the episode. ‘Binge drinking’ was used to define a single episode of drinking where reference was made to the patient having consumed significant quantities of alcohol, being drunk or exhibiting drunken behaviours in the absence of another drinking behaviour type being portrayed.

#### Disease/illness type

This described the patient’s primary presenting illness or disease rather than any pre-existing diagnoses or comorbidities. If patients exhibited more than one primary illness or disease they would be coded as having two illnesses. Where an individual presented with an injury, we did not determine the causal modality (e.g. accidental, self-inflicted, assault).

Coding categories were based on the disease categories used by Public Health England for classifying alcohol-related hospital admissions caused by health conditions wholly or partially attributable to alcohol.^3^^,^^32^ Attendances for diseases or illnesses that did not involve alcohol were coded as ‘non-alcohol-related illness’ (Supplementary material Table S2).

### Prevention

Any portrayal of prevention to reduce alcohol-related harms was coded. The dominant level was categorized as either primary, secondary or tertiary prevention. The type of prevention was then coded according to whether this involved medical (e.g. alcohol detoxification and/or specialist alcohol treatment services), lifestyle (e.g. reducing alcohol intake) and/or socio-structural (e.g. role of policy or legislation) approaches.

### Statistical analysis

Descriptive statistical analyses were undertaken using Microsoft Excel (Version 16.0.15). A Fisher’s exact test was also conducted using Stata SE (V.16) to compare the number of ARA episodes aired during the earlier (2011–2016) and later (2017–2022) years of the programme’s run. *P* values < 0.05 were considered statistically significant.

### Reliability and validity

No pre-existing data collection tools were identified from the literature to assess health event data in EE programmes. As a result, the author developed a bespoke data collection tool (spreadsheet for manual data entry) for the purposes of this study, using the coding categories presented above. This was piloted on a 20% sample of episodes to test its reliability and validity, with refinements made as appropriate. All data collection and analysis were undertaken by the author. A random sample of three episodes was initially coded by KW and HK individually, to ensure coding accuracy and interrater reliability. The two coders (KW and HK) measured this through percent agreement. Interrater agreement was high (83.33%); categories with lower agreement revolved around categorizing alcohol-related mental or behavioural disorders. Differences were resolved through discussion and KW continued to code the remaining episodes.

### Ethics

Ethical approval was granted by the Master’s in Public Health Ethics Committee, School of Medicine, and University of Nottingham.

## Results

Of 278 main series episodes, 27 met the inclusion criteria. Four episodes were unavailable via UK streaming services. The final sample consisted of 23 episodes, across 14 series, which were viewed online via All 4, Netflix and Prime Video. This identified 88 unique episode-functional patient ‘characters’. Of these, 38 (43.2%) presented with ARAs and formed the study cohort.

Episodes featuring ARAs accounted for 8.3% of all main series episodes broadcast between 2011 and 2022. Episodes portraying ARAs showed a decreasing trend over time, with more episodes featuring ARAs in the programme’s earlier years (20 episodes, 12.9%) versus the latter half (3 episodes, 2.4%, *P* = 0.002).

### Patient characteristics

Among the 38 patients with ARAs, 84.2% (n = 32) were main episode ‘characters’, with 63.2% male. ARAs occurred most in the youngest age group. The highest proportion of ARAs were observed in White ethnic groups (78.9%), with 71.1% of patients demonstrating confirmed alcohol use and 7.9% implied alcohol use. Alcohol-related settings were disclosed in 84.2% of ARAs. Pubs/bars/night clubs/‘nights out’ were the most commonly depicted setting type (65.6%) (Table 1).

**Table 1:** Characteristics for patients with ARAs in 24 Hours in A&E: by attendance type and total sample

	All	Alcohol use only	Alcohol related settings only	Combined presentations
	N = 38	N = 6	N = 11	N = 21
**Sex, n (%)**				
Female	14 (36.8%)	0 (0.0%)	5 (45.5%)	9 (42.9%)
Male	24 (63.2%)	6 (100%)	6 (54.5%)	12 (57.1%)
**Age group, n (%)**				
Young adult	28 (73.7%)	3 (50.0%)	8 (72.7%)	17 (81.0%)
Middle aged	8 (21.1%)	3 (50.0%)	3 (27.3%)	2 (9.5%)
Older adult	2 (5.3%)	0 (0.0%)	0 (0.0%)	2 (9.5%)
**Ethnic group, n (%)**				
Asian	2 (5.3%)	0 (0.0%)	0 (0.0%)	2 (9.5%)
Black	5 (13.2%)	1 (16.7%)	3 (27.3%)	1 (4.8%)
Mixed	1 (2.6%)	0 (0.0%)	1 (9.1%)	0 (0.0%)
White	30 (78.9%)	5 (83.3%)	7 (63.6%)	18 (85.7%)
Other	0 (0.0%)	0 (0.0%)	0 (0.0%)	0 (0.0%)
**Setting, n (%)** ^a^				
Private party	10 (31.3%)		7 (63.6%)	3 (14.3%)
Pub/bar/club	21 (65.6%)		4 (36.4%)	17 (81.0%)
Restaurant	1 (3.1%)		0 (0.0%)	1 (4.8%)
**Drinking behaviour, n (%)** ^b^				
Low risk	1 (3.7%)			
High risk	1 (3.7%)			
Alcohol dependence	4 (14.8%)			
Binge	19 (70.4%)			
Unknown	2 (7.4%)			

a n = 32 for ARAs involving alcohol related settings.

b n = 27 for confirmed alcohol use.

In patients with confirmed alcohol use, binge drinking (70.4%) was the most commonly portrayed drinking behaviour. Binge drinking was most frequently observed in young people (84.2%) and White ethnic groups (89.5%), with a slightly higher proportion of people who binge drink being males (52.6%) than females (47.4%). Alcohol dependence occurred exclusively in males and was most common in middle aged (75.0%) and White (75.0%) adults. However, when all patients with confirmed alcohol use are considered, binge drinking was the only drinking behaviour observed in females, older adults and Asian ethnic groups. Additionally, portrayals of alcohol dependence were featured in a comparatively higher proportion of Black (50.0%) and middle aged (60.0%) than young adults (5.0%), men (22.2%) and White ethnic groups (13.0%).

### Disease/illness types

Among the 38 patients with ARAs, 55 health events were observed across three disease/illness categories (24, 63.2% experienced only one health event).

The vast majority of health events (92.7%) observed in patients with ARAs involved conditions either partially or wholly attributable to alcohol. Accidents and injuries were the most commonly portrayed type of health event (72.7%). Followed by mental and behavioural disorders due to alcohol use (20.0%) and non-alcohol related illnesses (7.3%).

### Prevention

Prevention was portrayed in 15.8% (n = 6) of patients (secondary (50%), tertiary (50%)). Lifestyle measures (50%) were the most common prevention approach observed, followed by combined medical and lifestyle measures (33.3%) and medical measures alone (16.7%). No instances of primary prevention or socio-structural prevention were observed (Table 2).

**Table 2:** Characteristics of patients for whom prevention was portrayed

	Prevention portrayed	Row %
	N = 6	
**Sex, n (%)**		
Female	1 (16.7%)	7.1%
Male	5 (83.3%)	20.8%
**Age group, n (%)**		
Young adult	2 (33.3%)	7.1%
Middle aged	4 (66.7%)	50.0%
Older adult	0	0
**Ethnic group, n (%)**		
Asian	1 (16.7%)	50.0%
Black	2 (33.3%)	40.0%
Mixed	0	0%
White	3 (50.0%)	10.0%
Other	0	0%
**Drinking behaviour, n (%)**		
Low risk	0	0%
High risk	1 (16.7%)	100%
Alcohol dependence	3 (50.0%)	75.0%
Binge	2 (33.3%)	10.5%
Unknown	0	0%

Portrayals of medical measures typically referred to alcohol detoxification provided by specialist alcohol treatment services, while lifestyle measures involved advice to reduce alcohol intake.

When the total sample is considered, prevention was depicted nearly three times more frequently in men (20.8%) than women (7.1%) and seven times more frequently in middle aged patients (50.0%) than young adults (7.1%). A comparatively higher proportion of prevention events were depicted in ethnic minority groups, with prevention occurring in 50.0% of all Asian patients, 40.0% of Black patients, but only 10.0% of White patients (Table 2).

## Discussion

### Main findings of this study

Depictions of ARAs featured infrequently in main series episodes of *24 Hours in A&E* broadcast between 2011 and 2022 and demonstrated a largely decreasing trend over time. However, where ARAs were portrayed, they supported common cultural stigmas and stereotypes of alcohol use, being most common in men, young adults and White ethnic groups. Most portrayals featured presentations involving both alcohol use and alcohol-related settings. Pubs/bars/clubs/‘nights out’ represented the main alcohol-related setting type depicted, while binge drinking followed by alcohol dependence were the main drinking behaviours observed. Portrayals of ARAs encompassed a narrow spectrum of alcohol-related health consequences, with all health events depicted pertaining to three main disease/illness categories. Portrayals of prevention were featured in a minority of patients and did not support the known need for targeted interventions.

### What is already known on this topic

Depictions of ARAs only featured in 8.3% of *24 Hours in A&E* main series episodes, with less than half of the patients portrayed in these episodes experiencing ARAs. When compared with the results of a British cohort study, which found that 12–15% of A&E attendances were alcohol related,^4^ suggesting ARAs were underrepresented in *24 Hours in A&E.*

Additionally, significantly fewer episodes featuring ARAs were broadcast in the latter half of the programme’s run compared to its earlier years. There has been a well-publicised recognition during recent years of the substantial and avoidable burden on A&E resources created by heavy drinking.^4^^,^^33^^,^^34^ One explanation for the underrepresentation of ARAs, especially in more recent series, is that producers may have decreased alcohol-related content to avoid potential irresponsible coverage, to avoid perpetuating further the socially stigmatizing nature of alcohol. Alternatively, it may reflect the high priority of ratings figures (and therefore commissioning and advertising revenue) which require more dramatic ‘storylines’. While this under-representation could have positive impacts by helping to reduce inappropriate service use, previous research has shown that medical edutainment viewers typically underestimate the seriousness of under-portrayed health issues.^35^ Furthermore, there are potential impacts of alcohol-related stigma. It is acknowledged that higher levels of stigma are associated with perpetuating higher levels of consumption and lower help-seeking.^36^ Hiding potentially stigmatizing content around AUD and binge drinking can have complex effects on stigma. On one hand, reducing exposure to harmful stereotypes or language might help create a less judgmental environment. On the other, avoiding discussion of certain behaviours or issues might reinforce ignorance, allowing stigma to persist due to a lack of understanding.^37^ Effectively addressing stigma requires transparent, respectful communication that challenges harmful narratives while educating the public about the factors contributing to stigmatized behaviours and their broader societal implications.

This study found that ARAs were most common in men, young adults and White ethnic groups, consistent with the evidence-base. One UK study demonstrated that men accounted for 70% of ARAs.^38^ Furthermore, UK research indicates that ARAs are most common among younger age groups, with these patients typically being younger than those attending A&E for other health complaints.^4^^,^^39^ These findings contrast with research into other medical edutainment programmes, which found that men and younger patients were overrepresented in medical dramas.^26^^,^^40^ This suggests medical documentaries may provide more accurate age and gender portrayals than fictional programmes supporting persuasive effects on viewers due to perceptions of realism. These factors combined with the programme’s accessible nature, suggest it could be used to increase the reach of alcohol prevention messaging, particularly among groups with low health literacy and/or not reached by mainstream health communication channels.

Conversely, there are concerns that ethnic minority groups were underrepresented in ARA portrayals. People from Asian, Black, Mixed and Other ethnic groups comprise 42.1% of the population in London^41^ where *24 Hours in A&E* is filmed, but were only featured in 21.1% of ARA portrayals, suggesting ethnic minority groups were under-portrayed. Similar concerns regarding the underrepresentation of ethnic minority groups have been identified in medical dramas.^25^^,^^26^^,^^40^ This under-portrayal could attenuate the effectiveness of alcohol-related health messaging among ethnic minority audiences is of particular concern as ethnic minority groups are already recognized to have lower awareness of alcohol-related health risks.^42^

### What this study adds

Of concern was the underrepresentation of individuals with increasing and high-risk drinking behaviour when compared to reality. High-risk drinking was depicted in just 2.6% of patients and no instances of increasing risk drinking were observed. These findings contrast with UK research which estimates that 40.1% of A&E patients are increasing or high-risk drinkers,^43^ while another study found that individuals with increasing risk drinking comprised 54% of patients with ARAs.^39^ It was notable that no females with AUD were shown, whilst data suggests females account for 23% of alcohol dependence in England.^44^ Consequently, this under-portrayal could result in audiences underestimating the health impacts associated with these drinking behaviours as evidence has previously identified a dose–response relationship between alcohol portrayal and alcohol consumption.^45^ Given that nearly a quarter of the population is estimated to be drinking at these levels,^35^ this highlights a potential missed opportunity to increase public awareness of the health risks associated with these drinking behaviours.

Portrayals of ARAs encompassed a narrow spectrum of alcohol-related health consequences. The over-portrayal of accidents/injuries health events is a recognized challenge in medical EE programmes.^26^^,^^40^ While these graphic and dramatic conditions are appealing to viewers, this overrepresentation could lead to false perceptions of alcohol-related health risks—given the purported factual nature of the programme.

Prevention portrayals were minimal. All prevention depictions involved healthcare approaches with secondary or tertiary prevention and were mainly among patients with AUDs. Prevention was portrayed in all individuals with higher risk drinking behaviour, most commonly those with alcohol dependence, but only 10.5% of individuals with binge drinking behaviour. However, this is consistent with SBI clinical guidance, which focuses on harm reduction among increasing, high-risk and dependent drinking behaviour, with no specific action mandated for binge drinking behaviours in isolation.^5^^,^^7^^,^^8^

While the prevalence of prevention depictions seems low, there is some evidence that this reflects reality. Despite recommendations that SBI is conducted routinely, UK research has shown that alcohol screening is only undertaken in 61% of A&E patients, although nearly all those screening positive were offered appropriate help or advice for their alcohol problems.^46^ This was reflected in the prevention portrayals, with tertiary prevention involving medical approaches (e.g. alcohol detoxification) referenced in all prevention depictions among people with alcohol dependence, while secondary prevention involving lifestyle advice was depicted in the remaining patients.

Furthermore, most of these portrayals were consistent with the recommendations outlined in the clinical guidance,^7^^,^^8^ suggesting medical documentaries could provide accurate portrayals of healthcare interventions. Consequently, these programmes could be used to promote messaging to reduce unhealthy drinking behaviours and increase awareness of AUD interventions. This in turn could support help-seeking behaviours. Nevertheless, lifestyle advice alongside medical approaches featured in two thirds of prevention portrayals among people with alcohol dependence. This contradicts NICE guidance, which advises against non-alcohol specialists delivering brief advice to these patients.^7^ Similar concerns regarding the fidelity to clinical guidelines have been identified in medical dramas.^47^ As such, these inaccurate portrayals could result in false treatment expectations among viewers. Although, it should be noted this was observed in a minority of patients for whom prevention was depicted overall.

The findings present opportunities to improve clinical practice and to utilize streaming services as a platform to promote accurate health messaging. Although depictions of ARAs in the current study are specific to the British medical system, the streaming platforms hosting 24 Hours in A&E serve a global audience. Significant differences exist in the perceptions and acceptability of alcohol use internationally, alongside access to medical services and clinical guidance.^48^ The relevance of this study’s findings may not translate to the global viewership or wider international policy. However, recognition of the opportunity for medical documentaries to impact health behaviours and reinforce cultural stereotypes is universally applicable.

### Limitations of this study

To our knowledge this is the first study to quantitatively analyse depictions of alcohol-related health issues and prevention in a British medical documentary series. Development and validation of the bespoke data collection tool, alongside double coding of content, has ensured quality data collection. The author made all reasonable efforts to identify and code the totality of episodes featuring ARAs across the series and the potential limitation of a small sample size reflects reality. The focus on a single programme means that the findings may not be generalizable to other medical documentaries. However, *24 Hours in A&E* was considered an important programme to study given its substantial viewership and popularity among the British public. Additionally, patient demographic characteristics were not always explicitly stated, requiring assumptions to be made on the appropriate classification, which could have led to some coding misclassifications.

This study has shown mixed results regarding how accurately the programme portrays alcohol-related illnesses and prevention. Depictions of age, gender and prevention were largely accurate.

However, there are significant concerns regarding inaccurate and unrealistic portrayals, which could limit the programme’s utility as a health promotion tool. This includes the under-portrayal of ARAs and skewed ethnicity, diagnoses and drinking behaviour distributions. Together, these factors could result in false perceptions of alcohol-related health risks among audiences and persistence in unhealthy drinking behaviours. Consequently, there is a need for public health professionals to work with producers to support accurate portrayals of ARAs, while ensuring adequate coverage of alcohol-related health issues and prevention approaches. Furthermore, broadcasting standards could be strengthened to ensure content with an educational component is accurate and fair.

## Supplementary Material

Supplementary_files_fdae314

## Data Availability

Data is available on request via the corresponding author.

## Notes

**Karielle Webster**, MPH

**Holly Knight**, PhD

**Joanne R. Morling**, PhD
