# Supplementary material for: Quantifying the portrayal of alcohol-related A&E attendances and prevention in the British medical documentary series ‘24 hours in A&E’
Source: J Public Health (Oxf). 2025 Jan 12;47(2):309–16. doi: 10.1093/pubmed/fdae314 (PMC12123315; doi:10.1093/pubmed/fdae314)
Supplement: Supplementary_files_fdae314 [file supplementary_files_fdae314.docx]

**Supplementary material**

**Table S1.** **Episodes of 24 Hours in A&E portraying ARAs by series and viewing source**

| Series | Year first aired | Total number of episodes | Number of episodes depicting ARAs | Number of episodes included in analysis | Viewing source |
| --- | --- | --- | --- | --- | --- |
| 1 | 2011 | 14 | 5 | 3 | Prime Video |
| 2 | 2012 | 14 | 7 | 6 | All 4 |
| 3 | 2013 | 21 | 2 | 2 | All 4 |
| 4 | 2013 | 6 | 1 | 1 | Prime Video |
| 5 | 2014 | 8 | 2 | 1 | Prime Video |
| 6 | 2014 | 7 | 0 | 0 | - |
| 7 | 2014 | 8 | 1 | 1 | Prime Video |
| 8 | 2015 | 13 | 1 | 1 | Prime Video |
| 9 | 2015 | 8 | 1 | 1 | Netflix |
| 10 | 2015 | 24 | 1 | 1 | All 4 |
| 11 | 2016 | 11 | 1 | 1 | All 4 |
| 12 | 2016 | 21 | 2 | 2 | All 4 |
| 13 | 2017 | 12 | 0 | 0 | - |
| 14 | 2018 | 9 | 0 | 0 | - |
| 15 | 2018 | 18 | 1 | 1 | All 4 |
| 16 | 2018 | 6 | 1 | 1 | All 4 |
| 17 | 2019 | 8 | 0 | 0 | - |
| 18 | 2019 | 16 | 0 | 0 | - |
| 19 | 2019 | 5 | 0 | 0 | - |
| 20 | 2020 | 10 | 0 | 0 | - |
| 21 | 2020 | 4 | 0 | 0 | - |
| 22 | 2020 | 6 | 0 | 0 | - |
| 23 | 2021 | 8 | 0 | 0 | - |
| 24 | 2021 | 10 | 0 | 0 | - |
| 25 | 2021 | 3 | 0 | 0 | - |
| 26 | 2022 | 8 | 1 | 1 | All 4 |
| Specials | 2013-22 | 22 | 0 | 0 | - |
| Total | - | 300 | 27 | 23 | - |

**Table S2.** **Disease/illness categories and examples**

| Condition | Disease category and examples |
| --- | --- |
| Wholly attributable conditions | Alcoholic liver disease  Alcoholic cirrhosis of the liver  Alcoholic liver failure  Alcoholic hepatitis |
|  | Mental and behavioural disorders due to alcohol use  Alcohol dependence  AAI  Alcohol withdrawal (+/- seizures and or delirium)  Alcohol-related dementia (Wernicke-Korsakoff syndrome)  Other AUD  Other mental health disorder related to alcohol use (e.g. depression or anxiety) |
|  | Other wholly attributable conditions  Alcoholic cardiomyopathy  Alcoholic gastritis  Alcoholic polyneuropathy |
|  | Toxic effect of alcohol  Toxic effects of alcohol excluding mental and behavioural disorder due to alcohol use or where cause is not specified |
| Partially attributable conditions | Accidents and injuries  Burns  Head injuries (including facial and traumatic brain injuries)  Non-head injury (e.g. limb injuries or fractures)  Poisoning (e.g. accidental or intentional drug overdose) |
|  | Cancer  Colorectal cancer  Liver cancer  Oro-pharyngeal cancers |
|  | Cardiovascular disease  Cardiac arrhythmias  Ischaemic heart disease  Haemorrhagic or ischaemic stroke  Oesophageal varices |
|  | Digestive disease  Acute or chronic pancreatitis  Gall stones |
|  | Diseases of the nervous system  Epilepsy and status epilepticus |
|  | Infectious and parasitic diseases  Tuberculosis  HIV |
|  | Pregnancy and childbirth  Miscarriage |
|  | Respiratory infections  Pneumonia |
| Non-alcohol attributable conditions | Non-alcohol-related illness  Asthma  Inflammatory bowel disease  Multiple sclerosis  Sickle cell disease |
